# *Mycobacterium avium* subsp. *hominissuis* infection in horses with granulomatous enterocolitis – first report in Poland

**DOI:** 10.2478/jvetres-2025-0067

**Published:** 2025-12-10

**Authors:** Beata Nowicka, Wojciech Łopuszyński, Monika Krajewska-Wędzina, Anna Biazik, Magdalena Sobuś, Izabela Polkowska, Ewelina Szacawa

**Affiliations:** Department of Animal Surgery, 20-950 Lublin, Poland; Department of Pathomorphology and Forensic Veterinary Medicine, 20-950 Lublin, Poland; Department of Food Hygiene of Animal Origin, University of Life Sciences in Lublin, 20-950 Lublin, Poland; Department of Veterinary Surgery, Nicolaus Copernicus University in Toruń, 87-100 Toruń, Poland; Department of Diagnostics and Clinical Sciences, Institute of Veterinary Medicine, Nicolaus Copernicus University in Toruń, 87-100 Toruń, Poland; Department of Bacteriology and Bacterial Animal Diseases, National Veterinary Research Institute, 24-100 Puławy, Poland

**Keywords:** abdominocentesis, enterocolitis, intractable equine diarrhoea, MAH, MALDI-TOF

## Abstract

**Introduction:**

Gastrointestinal mycobacteriosis in horses is difficult to diagnose because of the pathogen’s intracellular nature and the non-specific clinical symptoms. Effective accurate diagnosis facilitates prognosis and treatment. Current diagnostic procedures and methods of collecting material do not permit definitive antemortem diagnosis. However, culturing, acid-fast bacilli staining, histopathology, PCR and immunological marker evaluation may prove useful.

**Material and Methods:**

Three horses were admitted to a clinic for intensive care and a final diagnosis. Physical examination and additional tests were performed. Unfavourable prognoses and lack of treatment response prompted euthanasia decisions. Necropsy was performed, as were histological, microbiological and molecular investigations.

**Results:**

The clinical condition of the animals deteriorated despite therapy. Two horses were euthanised when they did not respond to treatment and had poor prognoses. Intestinal mycobacteriosis caused by *Mycobacterium avium* subsp. *hominissuis* was diagnosed postmortem using laboratory investigations. One horse’s diagnosis was established antemortem by cytological and microbiological examination of biopsy material from an abdominocentesis, and this animal was also euthanised because of its poor prognosis.

**Conclusion:**

Mycobacteriosis should be considered in the differential diagnosis of chronic debilitating equine diarrhoea in addition to rhodococcosis, lawsoniosis, salmonellosis, gastric ulcers and food intolerance. Peritoneal fluid obtained by abdominocentesis proved to be an effective diagnostic method for microbiological and molecular identification of *Mycobacterium avium* subsp. *hominissuis* in horses with suspected enteric mycobacteriosis and concomitant ascites.

## Introduction

Mycobacterial infections in horses are generally rare and require a complex diagnostic approach (4, 5, 9, 15). Advances in molecular biology, gene sequencing and classification of pathogens have made possible the identification of many species and subspecies within the *Mycobacterium* genus. Most mycobacteria are grouped into the two most dominant complexes, namely *M. tuberculosis* complex (MTBC) and *M. avium* complex (MAC) (18, 36). The first largely comprises the mycobacteria that cause tuberculosis in both humans and animals including horses. Tuberculosis was the world’s second leading cause of death from a single infectious pathogen in humans in 2022 (36). The second complex, which is responsible for mycobacteriosis, includes two species: *M. avium* and *M. intracellulare*, of which the first is split into four subspecies: *M. avium* subsp. *avium, M. avium* subsp. *silvaticum, M. avium* subsp. *paratuberculosis* and *M. avium* subsp. *hominissuis* (MAH). All members have worldwide distribution and zoonotic potential (24, 25). The bacteria of the MAC are widespread in the environment, being found mainly in soil and water reservoirs, both in natural and in tap water. The subspecies other than *M. avium* subsp. *paratuberculosis* cause mycobacterial infections in birds (25, 28). That subspecies is responsible for Johne’s disease (paratuberculosis) in ruminants, mainly cattle (7, 8, 23) and MAH causes human, swine, wild animal and horse infections (14, 24).

The likely evolutionary carriers of MAH, ubiquitous free-living amoebae and other environmental hosts, populate the environment which this opportunistic pathogen typically inhabits (32). It is entirely probable that interactions between MAH and free-living amoebae have exerted selective pressure on the bacterium to acquire survival mechanisms (also used against macrophage predation) (1, 27). In macrophage predation, the pathogenic bacterium is located inside phagosomes and as MAH, it has been shown to exit them to infect other cells. Its adaptation to the hostile intraphagosomal environment is through multiple virulence mechanisms. There is an MAH ability to switch macrophages to a non-inflammatory phenotype (1, 31). The bacterium produces a multitude of antioxidant enzymes, including catalase, superoxide dismutase and alkyl hydroxide reductase, and produces oxidative repair enzymes including thioredoxin and thioredoxin reductase. These defences are regulated by several oxidative sensors that induce transcription of antioxidant and oxidative repair enzymes and biofilm-promoting genes. These expressions induce physiological changes that allow MAH to survive the leukocyte-generated oxidative stress (1). The aim of this article is to describe the clinical course of the disease, gross and microscopic findings and the results of laboratory investigation in three horses diagnosed with gastrointestinal tract mycobacteriosis. To the best of the authors’ knowledge, this is the first description of gastrointestinal mycobacteriosis caused by MAH in horses in Poland.

## Material and Methods

### Animals

The three horses from the Lubelskie province included in the study were treated by farm veterinarians in the initial stage of the disease. Subsequently, treatment brought no effects and the horses’ health was deteriorating; therefore, the animals were referred to a university veterinary clinic for intensive care and a definitive diagnosis. The clinical data and the results of laboratory investigations are summarised in Table 1.

**Table 1.** The clinical data of horses suffering granulomatous enterocolitis and the results of laboratory investigations

Clinical and laboratory data	Horse No. 1	Horse No. 2	Horse No. 3
Age	3 months	10 months	3 years
Sex	Male	Male	Female
Duration of illness	2 months	7 months	1.5 years
Symptoms:			
Diarrhoea	+	+	+
Weight loss	+	+	+
Gastric ulcers	+	+	+
Serum biochemistry:			
Hypoalbuminaemia	- (10.7 g/L)	- (12.1 g/L)	- (9.8 g/L)
Parasitological findings after examination of faecal samples	-	Parascaris univalens, Cryptosporidium sp. and Giardia sp.	-
Ultrasonographic findings	Thickened intestinal wall	Hyperechoic mass in the lower left area (12-14 intercostal spaces), presence of free fluid in the abdominal cavity, thickened small intestinal wall	Free fluid present in the abdominal cavity, thickened intestinal wall

All three horses were warmbloods. The duration of clinical symptoms before admission to the clinic ranged from 2 months to 1.5 years. The horses all had histories of anorexia, weight loss and long-lasting diarrhoea. Hypoalbuminaemia was also noted in all of them. An abdominocentesis and a biopsy of the rectal mucosa from horse No. 3 were performed and the material was stained with the Ziehl–Neelsen (ZN) method. The result was positive for abdominal fluid and negative for the presence of acid-fast bacteria in rectal mucosa sample. Similarly, the results of PCR and the ELISA from the collected materials (blood and faecal material) were negative in all cases.

### Necropsy and histopathology

The necropsy examination was performed with the horse placed on its right side, in accordance with generally accepted principles. Samples of the small and large intestine, ileocaecal and mesenteric lymph nodes, and lesioned liver and heart tissue were collected during necropsy and immediately fixed in 10% neutral buffered formalin for 24 h, then automatically dehydrated with graded alcohol solutions, cleared with acetone and xylene and embedded in paraffin blocks in a TP-1020 tissue processor (Leica, Nussloch, Germany). Histology sections 4-μm-thick were sliced on an SM2000-R sledge microtome (Leica). Then, after dewaxing in xylene and rehydration through a graded alcohol series to distilled water, tissue sections were stained with haematoxylin and eosin and ZN. Evaluation was made using an Eclipse E-600 light microscope (Nikon Instruments, Tokyo, Japan) and images captured with a DS-Fi1 digital camera (Nikon Instruments) and a personal computer with NIS-Elements BR-2.20 image analysis software (Laboratory Imaging, Praha, Czech Republic).

### Mycobacterial culture

In the culture study, approximately 3 g of examined small intestine tissue was shredded and placed in sterile filter bags (Interscience, Schaffhausen, Switzerland). A 15-mL aliquot of 5% oxalic acid (C_2_H_2_O_4_) was added to the sample placed in the sterile bag and the bag’s contents were homogenised for 3 min. The filtrate was then poured into a Falcon tube (Thermo Fisher Scientific, Waltham, MA, USA) and incubated for 20 min at 37°C (±2°C). After this step, it was centrifuged for 10 min at 4000 × *g*, the supernatant was removed and the pellet was washed twice in saline solution (0.90% w/v) and centrifuged again for 10 min at 3,500 × *g*. Part of the obtained sediment was plated on three Stonebrink and three Petragniani media (BioMaxima, Lublin, Poland) and incubated for six weeks at 37°C (±2°C) with weekly readings. The remainder of the obtained sediment was tested in a Mycobacteria Growth Indicator Tube automated liquid culture system (MGIT; Becton Dickinson, Frankin Lakes, NJ, USA) at the same time. The sediment was prepared using the MycoPrep kit (Becton Dickinson) used to decontaminate samples containing mycobacteria according to the manufacturer’s protocol. The prepared samples were inoculated into a three liquid medium and incubated at 37°C.

### DNA isolation and real-time PCR

Mycobacteria are pathogenic to humans; therefore they must be inactivated before proceeding with the isolation of their nucleic acids. In this operation, the thermal method was used, and the sample was inactivated at 100°C for 10 min. A real-time PCRwas used to quickly confirm or exclude the presence of MTBC bacteria in the examined tissues. The primers used were specific for the insertion sequence (IS) IS6110, which is found exclusively in the members of the MTBC (Table 2). Their sequences were obtained from the European Reference Laboratory for Bovine Tuberculosis in Madrid, Spain. In the first stage of the research, DNA was isolated from a fragment of mesenteric lymph node tissue. For this purpose, the DNeasy Blood & Tissue kit (Qiagen, Hilden, Germany) was used and the manufacturer’s instructions were followed. After extraction, 5 μL of DNA was taken and used for the reaction (Table 3).

**Table 2.** Starter and probe sequences used in the reaction to screen equine tissue samples for *Mycobacterium tuberculosis* complex species

Name	Sequence 5′–3′
Starter F6110	GGT AGC AGA CCT CAC CTA TGT GT
Starter R6110	AGG CGT CGG TGA CAA AGG
Pr6110	FAM-CAC GTA GGC GAA CCC-MGB NFQ

1 F – forward; R – reverse; Pr – probe

**Table 3.** Components in the PCR reaction to screen equine tissue samples for *Mycobacterium tuberculosis* complex species

Reagent	Concentration	Volume (μL)
Nuclease-free water	-	7.5
QuantiFast mix	5×	5
Assay internal control	10×	2.5
Internal control DNA	10×	2.5
F6110	5 pmol/μL	1
R6110	5 pmol/μL	1
Pr6110	5 pmol/μL	0.5
DNA	-	5
Total volume	25	

1 F – forward; R – reverse; Pr – probe

### Strain species identification

The species identification of the strain was made with the Genotype Mycobacterium CM (common mycobacteria) test (Hain Lifescience, Nehren, Germany) in accordance with the manufacturer’s protocol. In order to be certain of valid results, two methods of strain identification were used. The method used to confirm the results of the Hain Lifescience CM test was matrix-assisted laser desorption/ionisation–time-of-flight mass spectrometry ((MALDI-TOF MS) with a Biotyper System (Bruker, Billerica, MA, USA). For this purpose, an amount of bacterial biomass corresponding to two full volumes of a calibrated 1 μL inoculation loop was added to a test tube containing 50 μL of trifluoroacetic acid. The prepared sample was incubated for 30 min at room temperature, and then it was diluted tenfold by adding 450 μL of sterile distilled water. A 1 μL aliquot of the diluted mixture was applied to a MALDI plate. After the applied sample was dry, 1 μL of matrix was added and the solution was analysed.

In order to identify the subspecies of the *M. avium* strain, next-generation sequencing was performed. Chemical lysis by cetyltrimethylammonium bromide was used for DNA isolation. After quality control of the extracted DNA, DNA libraries were prepared with a Nextera XT DNA Library Preparation Kit (Illumina, San Diego, CA, USA). The samples were sequenced on the Illumina MiSeq platform using the MiSeq Reagent Kit v3 (Illumina). Fragments of DNA encoding IS 901, IS1245, IS311 and 16S rRNA were analysed. For this purpose, the National Center for Biotechnology Information basic local alignment search tool was used, in which the obtained sequences were compared with all sequences available in the GenBank database.

## Results

Haematological and biochemical abnormalities included leukocytosis, hypoalbuminaemia, and hyperfibrinogenaemia. In all horses, despite attempts at therapy, clinical symptoms worsened after a prolonged period of illness. Two of the three horses were euthanised because there was no response to symptomatic treatment and they had poor prognoses. The diagnosis of intestinal mycobacteriosis was made postmortem through laboratory investigations. In one horse (No. 3), the final diagnosis was established after examination of material collected during abdominocentesis. Acid-fast bacilli were found in a bacterioscopic preparation stained according to the Ziehl-Neelsen method. After a definitive diagnosis was made, the horse was also euthanised.

### Necropsy and histopathological examination

**Fig. 1.**
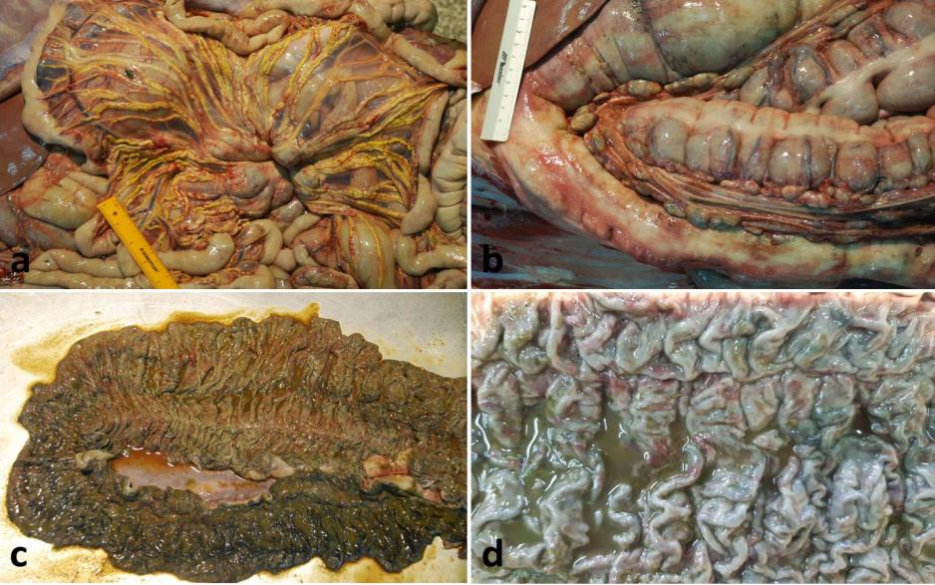
Gross pathology photographs of tissue sections from Polish horses with granulomatous enterocolitis. a – Markedly enlarged mesenteric lymph nodes, the segment of the small intestine drained by thick, dilated mesenteric lymphatic vessels (mesenteric lymphangitis/lymphangiectasia); b – Large colon (sternal/diaphragmatic flexure): colonic lymph nodes enlarged to a size of 1–3 cm in diameter; c – Large colon (pelvic flexure) with diffusely, markedly thickened mucosa; d – Large colon, irregularly transversely folded mucosal rugae with multifocal superficial erosions

**Fig. 2.**
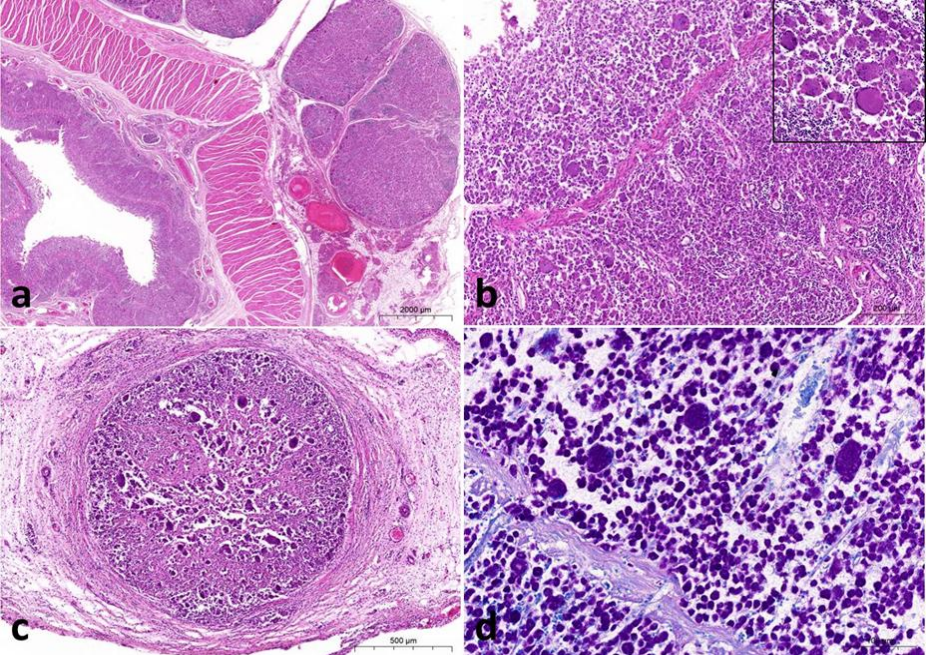
Micrographs of tissue samples from Polish horses with granulomatous enterocolitis. a – Haematoxylin and eosin (HE)-stained cross section through a large colon wall and adjacent lymph nodes. Massive granulomatous inflammatory infiltrates in the mucosa and submucosa and in the adjacent lymph node parenchyma. Remnants of lymphoid follicles visible in the cortex. Scale bar = 2000 μm; b – HE-stained lamina propria and submucosa of the large colon widely infiltrated by epithelioid macrophages and aggregates of lymphocytes and plasma cells; multinucleated giant cells with peripherally located nuclei (insert). Scale bar = 200 μm; c – HE-stained cross section through a mesenteric lymphatic vessel occluded by epithelioid macrophages, multinucleated giant cells and fewer lymphocytes and plasma cells. Scale bar = 500 μm; d – Ziehl–Neelsen-stained large colon with numerous macrophages in the lamina propria and submucosa containing intracellular acid-fast bacteria. Scale bar = 100 μm

At necropsy horses Nos 1 and 2 showed variable but severe wasting. Faecal contamination of the hind legs and perianal region was consistent with the clinical history of diarrhoea. The major gross findings in both horses included increased amounts of turbid, straw-coloured fluid in the peritoneal cavity (ascites); enlarged mesenteric lymph nodes associated with the ileum, large colon and caecum; marked thickening of the mesenteric lymphatic vessels; and significant thickening of the wall of the ileum, large colon and caecum (Fig. 1a and 1b). In addition, horse No. 1 had two large granulomas in the liver measuring 5 cm in diameter and a single large granulomatous lesion measuring 8 cm in diameter in the wall of the left atrium. The mucosa of the distal segment of the small intestine and the entire surface of the large colon and caecum was severely thickened, rough, oedematous and folded, with multiple superficial small erosions (Fig. 1c and 1d). Multiple full-thickness samples of the small and large intestines, ileoceacal lymph nodes, mesenteric lymphatic vessels and granulomas from the livers and hearts were examined microscopically. Histopathological examination of the intestines revealed numerous large macrophages with abundant foamy cytoplasm (epithelioid macrophages) arranged in dense sheets diffusely and markedly infiltrating the lamina propria and submucosa. They were separating hyperplastic crypts widely and replacing them. Villi of the ilium were blunted and fused and formed prominent mucosal rugose folds. Similarly, in the colon and caecum, focal ulcerations were present at the apex of the wrinkled folds and a massive infiltrate composed of epithelial macrophages admixed with low numbers of lymphocytes, plasma cells, eosinophils and multinucleated giant cells was in the process of replacing and expanding the lamina propria, muscularis and subserosal adventitia (Fig. 2a and 2b). The intestinal lymph node parenchyma was almost completely effaced by aggregates of foamy macrophages and multiple multinucleated giant cells, which extended into the cortex and medulla. The remaining lymphoid follicles were visible only in the subcapsular cortex. The lumen of the dilated mesenteric lymphatic vessels was almost completely occluded by clusters of epithelioid macrophages and inflammatory cells (Fig. 2c). The presence of identical inflammatory infiltrates in the wall of the caecum and satellite lesions in the liver and heart were confirmed. Histological samples of intestinal mucosa and lymph nodes stained with Ziehl–Neelsen revealed numerous acid-fast bacilli within the cytoplasm of macrophages (Fig. 2d). The gross and microscopic findings were consistent with severe, diffuse, chronic non-caseous granulomatous enterocolitis and typhlitis, granulomatous mesenteric lymphadenitis and mesenteric lymphangiectasia with numerous acid-fast intracellular bacilli.

### Laboratory diagnosis

Culture examination and molecular findings confirmed MAH infection. Visible bacterial growth was obtained in the third week of culture incubation, and was twice as abundant on Stonebrink medium as on Petragnani medium. The MGIT system indicated the presence of *Mycobacterium* in all tubes. The results of the real-time PCR clearly indicated the absence of tuberculous mycobacteria in the examined tissue, and confirmed that the isolated strain belonged to the nontuberculous mycobacteria species.

The GenoType CM test results clearly indicated that the analysed strain belonged to the *M. avium* species. In the final phase of the test, a clear position 4 of the test strip was seen, which is characteristic only for this species of bacteria. This was confirmed by the results of the analysis of the cell proteins of the tested strain using MALDI-TOF MS. Based on DNA sequencing, the strain was determined to be *Mycobacterium avium* subsp. *hominissui*s (Table 4).

**Table 4.** Matrix-assisted laser desorption/ionisation–time-of-flight mass spectrometry (MALDI-TOF MS) results of *Mycobacterium* species identification in tissue samples from Polish horses with granulomatous enterocolitis

No.	MALDI-TOF MS results			
	Species	Points	Probability	Symbols/colour
Horse No. 1	*Mycobacterium avium* subsp. *hominissuis*	≥2,300	Highly probable	+++/green
Horse No. 2	*Mycobacterium avium* subsp. *hominissuis*	≥2,300	Highly probable	+++/green
Horse No. 3	*Mycobacterium avium* subsp. *hominissuis*	≥2,300	Highly probable	+++/green

1 MALDI-TOF MS – matrix-assisted laser desorption/ionisation–time-of-flight mass spectrometry

## Discussion

Infections caused by *Mycobacterium avium* are described in humans and animals with increasing frequency (10, 15, 25). Disseminated alimentary mycobacteriosis has been described in horses, but most cases were diagnosed postmortem similarly to the cases of horses Nos 1 and 2 in this article (3, 19, 26, 30). Gastrointestinal mycobacteriosis caused by MAH was also recorded in two captive black howler monkeys (*Alouatta caraya*) in Polish zoos (6). The monkeys suffered from recurrent diarrhoea, and, as in the case of the described horses, the diagnosis was made postmortem (6). *In vivo* diagnosis of MAC infection in horses remains difficult and problematic because of the diversity of clinical signs as well as the low sensitivity and specificity of the available diagnostic tests for MAC or MTBC. The clinical signs in horses with disseminated gastrointestinal mycobacteriosis are similar to those caused by *Salmonella* spp., *Rhodococcus equi, Lawsonia intracellularis*, and equine rotaviruses and coronaviruses (33). They typically include diarrhoea, inappetence, lethargy and weight loss that often resemble protein-losing enteropathies commonly associated with inflammatory bowel disease (11, 15). All the cases we described had these symptoms. In addition, gastric ulcers were found in all of our cases, which could also have masked the underlying disease (17). The tuberculin skin test, commonly used in cattle, may give positive results in up to 70% of clinically healthy horses; therefore it is not considered a reliable diagnostic approach. Serology also cannot offer an effective means of diagnosing mycobacterial infection in horses, because the serological response to this infection has not been fully elucidated (5, 13). Mycobacterial infections cause granulomatous inflammation, which indicates a diagnostic possibility in collecting material by biopsy from the affected organs. Liver biopsy has been suggested as a diagnostic procedure in horses if the differential diagnosis includes MAC infections or granulomatous enteritis, especially when serum liver-specific enzyme activity is high (16). The recommended tests to confirm the diagnosis of suspected intestinal mycobacteriosis include biopsy of the rectum and distal colon and histological visualisation to detect acid-fast bacilli with specific preparations such as ZN stain (22). However, rectal biopsy evaluation in five horses suspected of having gastrointestinal mycobacteriosis revealed mild multifocal neutrophilic inflammation or mild granulomatous proctitis, but a ZN stain for mycobacteria was positive in only one case (19). In diagnostically difficult cases, exploratory laparotomy was also performed. Intraoperative findings included multifocal tuberculous lesions in the serosa of the dorsal colon and small intestine and on the ventral surfaces of the diaphragm. Histological examination of biopsies taken from these sites revealed granulomatous inflammation, although no bacteria were found (26). We did not perform these procedures on horses Nos 1 and 2 because of their poor health condition and the owners’ decision to euthanise them.

Mycobacteria are intracellular organisms, which makes recognition of them as aetiological agents difficult (18, 19). Methods based on DNA and RNA are used to identify *M. avium* complex in food, soil and animal-tissue samples (21). The diagnostic methods of choice are molecular identification by PCR and the determination of increasing antibody titres (7, 14, 36). The achievement of identification of mycobacteria in the faeces of healthy horses as well as sick ones is a consideration against faecal examination as an effective diagnostic method (16, 20). Laboratory tests of faeces and a biopsy of the rectum of horse No. 3 were performed, but both the PCR and ELISA tests gave negative results. In this case, fluid collected from the abdominal cavity made it possible to obtain a diagnosis based on a microbiological investigation and PCR. In humans, the same diagnostic procedure for mycobacterial infections has been described and is considered useful (34).

Although horses are considered a species resistant to mycobacterial infections compared with other domestic and wild animal species, many factors can compromise their natural immunity. The development of *M. avium* subsp. *hominissuis* infection in the gastrointestinal tract may be caused by certain immunodeficiencies related to genetic predisposition (14). Pituitary dysfunction and parasitic infestation were also mentioned as risk factors for mycobacterial infection (30). Based on the available medical history for the patients we described, horses Nos 2 and 3 were born by artificial insemination with frozen semen from the same stallion. However, we do not have clear evidence that equine semen can be the source of mycobacteriosis, so we can only assume this possibility. Cases of transmission of mycobacterial infection through semen among other species have been reported (2, 8, 12, 35).

In our cases, the postmortem examination and microscopic investigations confirmed lesions consistent with disseminated intestinal granulomatous inflammation (lepromatous type), similar to those in horses described in the literature (3, 19, 26, 29). *Mycobacterium avium* subsp. *hominissuis* can also cause lymphoplasmacytic enteritis and granulomatous colitis in horses (15, 19). Similarly, based on the histopathology and microbiological examinations of the three horses in this article, MAC can be considered a possible aetiological factor in horses suspected of having chronic inflammatory infiltrative enteritis. Current scientific reports clearly indicate that mycobacterial infections in horses have unfavourable prognoses (3, 5, 9, 17). In humans MAC infections occur mainly in people with functional predispositions or weakened immunity. These infections are most often associated with secondary immunodeficiencies caused by human immunodeficiency virus, tumour necrosis factor α antagonist therapy, immunosuppressive therapy after transplantation, lung diseases such as cystic fibrosis and chronic obstructive pulmonary disease or other systemic diseases. Risk factors for MAC infection also include gastroesophageal reflux disease, vitamin D deficiency, rheumatoid arthritis and low body mass index. It should be noted that the disease can also develop in immunocompetent individuals (10). The greatest risk of infection for veterinary personnel is likely to occur during necropsy of infected horses, and awareness of these potential hazards remains extremely important.

## Conclusion

*Mycobacterium avium* complex should be considered in the differential diagnosis of chronic debilitating diarrhoea in horses in addition to common diseases caused by *Rhodococcus equi* and *Lawsonia intracellularis* infections, gastric ulcers, food intolerance and other factors. Abdominocentesis might be the method of choice, useful in more instances in the diagnosis of gastrointestinal mycobacteriosis in horses. Consideration should be given to MAC as a potential source of infection for horses bred together as well as for veterinary personnel and immunocompromised people who come into contact with potential carriers.
